# Subthalamic Nucleus Deep Brain Stimulation Modulate Catecholamine Levels with Significant Relations to Clinical Outcome after Surgery in Patients with Parkinson’s Disease

**DOI:** 10.1371/journal.pone.0138462

**Published:** 2015-09-22

**Authors:** Tatsuya Yamamoto, Tomoyuki Uchiyama, Yoshinori Higuchi, Masato Asahina, Shigeki Hirano, Yoshitaka Yamanaka, Satoshi Kuwabara

**Affiliations:** 1 Department of Neurology, Graduate School of Medicine, Chiba University, Chiba, Japan; 2 Department of Neurosurgery, Graduate School of Medicine, Chiba University, Chiba, Japan; 3 Department of Neurology, Dokkyo Medical College, Tochigi, Japan; Mayo Clinic, UNITED STATES

## Abstract

**Aims:**

Although subthalamic nucleus deep brain stimulation (STN-DBS) is effective in patients with advanced Parkinson’s disease (PD), its physiological mechanisms remain unclear. Because STN-DBS is effective in patients with PD whose motor symptoms are dramatically alleviated by L-3,4-dihydroxyphenylalanine (L-DOPA) treatment, the higher preoperative catecholamine levels might be related to the better clinical outcome after surgery. We aimed to examine the correlation between the preoperative catecholamine levels and postoperative clinical outcome after subthalamic nucleus deep brain stimulation. The effectiveness of STN-DBS in the patient who responded well to dopaminergic medication suggest the causal link between the dopaminergic system and STN-DBS. We also examined how catecholamine levels were modulated after subthalamic stimulation.

**Methods:**

In total 25 patients with PD were enrolled (Mean age 66.2 ± 6.7 years, mean disease duration 11.6 ± 3.7 years). Mean levodopa equivalent doses were 1032 ± 34.6 mg before surgery. Cerebrospinal fluid and plasma catecholamine levels were measured an hour after oral administration of antiparkinsonian drugs before surgery. The mean Unified Parkinson’s Disease Rating Scale scores (UPDRS) and the Parkinson’s disease Questionnaire-39 (PDQ-39) were obtained before and after surgery. Of the 25 patients, postoperative cerebrospinal fluid and plasma were collected an hour after oral administration of antiparkinsonian drugs during on stimulation at follow up in 11 patients.

**Results:**

Mean levodopa equivalent doses significantly decreased after surgery with improvement in motor functions and quality of life. The preoperative catecholamine levels had basically negative correlations with postoperative motor scores and quality of life, suggesting that higher preoperative catecholamine levels were related to better outcome after STN-DBS. The preoperative plasma levels of L-DOPA had significantly negative correlations with postoperative UPDRS- III score in off phase three months after STN-DBS. The preoperative cerebrospinal fluid (CSF) 3,4-dihydroxyphenylacetic acid (DOPAC) and 5-hydroxytryptamine (5-HT) levels had significantly negative correlations with postoperative UPDRS- III score in off phase one year after STN-DBS and the preoperative CSF homovanilic acid (HVA) levels had significant negative correlations with postoperative UPDRS- III score in on phase three months after STN-DBS. In PDQ-39 SI (summary index), preoperative plasma dopamine (DA) level had significantly negative correlations with postoperative PDQ-39 SI one year after STN-DBS suggesting that higher preoperative plasma DA level resulted in better quality of life (QOL) one year after STN-DBS. The stepwise multiple linear regression study revealed that higher preoperative plasma HVA levels had negative influence on the postoperative motor symptoms (i.e., increase in the score of UPDRS), whereas higher preoperative CSF L-DOPA levels had positive influence on the postoperative motor symptoms and QOL (decrease in the score of UPDRS and PDQ-39 SI) The catecholamine levels were not significantly reduced postoperatively in 11 patients despite the significant reduction in levodopa equivalent doses. Unexpectedly, CSF HVA levels significantly increased from 0.00089±0.0003 ng/μl to 0.002±0.0008 ng/μl after STN-DBS.

**Conclusion:**

The preoperative catecholamine levels might affect the postoperative motor symptoms and quality of life. The catecholamine levels were not significantly reduced postoperatively despite the significant reduction in levodopa equivalent doses.

## Introduction

Subthalamic nucleus deep brain stimulation (STN-DBS) is the preferred surgical therapy in patients with advanced Parkinson’s disease (PD) [1]. However, its physiological mechanisms remain unclear [2–4]. STN-DBS is effective in patients with PD whose motor symptoms are dramatically alleviated by L-3,4-dihydroxyphenylalanine (L-DOPA) treatment [1], suggesting that the higher preoperative catecholamine levels might be related to the better clinical outcome after surgery. However, we do not know the relationship between the preoperative catecholamine levels and the outcome of STN-DBS. Therefore, one of the purposes of this study is to clarify the relationship between the preoperative catecholamine levels and postoperative motor symptoms, cognitive functions and quality of life (QOL).

The experimental studies proposed that STN-DBS affects neurotransmitter release in the basal ganglia [5–9]. Although, STN-DBS works even in the absence of dopaminergic medication, the effectiveness of STN-DBS in the patient who responded well to dopaminergic medication suggest the causal link between the dopaminergic system and STN-DBS. Some studies have examined the effect of STN-DBS on striatal dopamine (DA) release [8,9]. Although positron emission tomography (PET) measurements of [^11^C] raclopride uptake during STN-DBS failed to demonstrate any change in striatal DA in humans [10], several studies using a rodent model of PD revealed an increased striatal DA release with STN-DBS [8,9,11–14]. The effect of STN-DBS on the DA metabolites such as 3,4-dihydroxyphenylacetic acid (DOPAC) and homovanillic acid (HVA) are also unknown in human. The second purpose of this study is to examine how plasma and CSF levels of catecholamine (including DA and serotonin and their metabolism) change after STN-DBS. We also aimed to examine serotonin and its metabolites because serotonergic raphe nuclei projecting to striatum are known to be degenerated and several studies have examined CSF serotonin levels in Parkinson’s disease [15].

## Patients and Methods

### Patients

Between December 2009 and October 2014, 25 patients undergoing bilateral STN-DBS implantation at the Chiba University Hospital were enrolled in the study. Patients were diagnosed with PD based on the United Kingdom (UK) Parkinson’s Disease Society Brain Bank clinical diagnostic criteria [16], and had reported medication-resistant motor fluctuations and complications. The clinical background of the patients is listed in Table 1. Prior to enrolment in the study, patients had been treated with antiparkinsonian medications and were taking L-DOPA, decarboxylase inhibitors (DCIs), DA agonists, selegiline and entacapone; none took anticholinergics immediately before or during the study. The motor functions in the on and off phases were evaluated using the Unified Parkinson’s Disease Rating Score (UPDRS) Part II and Part III, before and after STN-DBS. Quality of life was assessed with the Parkinson’s Disease Questionnaire-39 (PDQ-39) summary index (SI) before and after STN-DBS. The cognitive functions were evaluated by mini mental state examination (MMSE) and frontal assessment battery (FAB). The UPDRS and the PDQ-39 SI were assessed with on stimulation after STN-DBS. Postoperative clinical evaluations were performed after 3 months and 1 year. The levodopa equivalent dose (LED) of antiparkinsonian medications was calculated as previously described.

**Table 1:** Levodopa equivalent dose (LED), motor (UPDRS-II and III in on and off phase), cognitive (MMSE and FAB) and quality of life (PDQ-39 SI) before and after (3 months and 1 year) STN-DBS.

	Before STN-DBS	3 months after STN-DBS	1 Year after SYN-DBS	P value (Before vs 3M after)	P value (Before vs 1 Y after)
LED (mg)	1032.44±34.60	620.46±42.31	588.10±41.24	p<0.001	p<0.001
Part2 off	22.48±1.44	11.66±2.44	13.10±3.92	p = 0.017	p = 0.20
Part2 on	8.20±1.16	8.04±1.37	8.21±1.47	p = 0.86	p = 0.43
Part3 off	44.38±2.36	22.40±3.02	25.05±3.78	p = 0.0006	p = 0.0036
Part3 on	19.84±1.48	13.46±1.67	15.95±1.98	p = 0.0038	p = 0.0096
MMSE	28.11±0.40	28.18±0.44	28.92±0.393	p = 0.08	p = 0.25
FAB	16.07±0.55	14.37±0.87	16.08±0.58	p = 0.08	p = 0.47
PDQ-39 SI	33.92±2.96	25.61±2.50	26.97±3.33	p = 0.032	p = 0.40

### Measurements of catecholamine levels in plasma and CSF

Plasma and CSF samples were collected an hour after oral administration of antiparkinsonian drugs (100mg of L-DOPA/DCI) in 25 patients before surgery. All patients agreed to the lumbar puncture procedure for evaluation before surgery. Of the 25 patients, CSF samples were collected in 11 patients who agreed to the lumbar puncture procedure at the follow up appointments, postoperatively. Postoperative plasma collection was only performed in 11 patients who agreed to the lumbar puncture procedure. The lumbar puncture at the follow up appointments was optional examination due to its invasive characteristics. Postoperative lumbar puncture was performed after 3 months in 6 patients, after 6 months in 1 patient, and after 1 year in 3 patients. The plasma and CSF samples were stored at −80°C. and catecholamine levels were analyzed using high-performance liquid chromatography (HPLC). Samples were pretreated with alumina extraction before measurements. Monoamines were detected with an equipped electrochemical detector system (HTEC500; Eicom). The mobile phase involved 0.1 M citric acid–0.1 M sodium acetate (pH 3.9) containing 140 mg/L sodium 1-octane sulfonate, 5 mg/L EDTA-2Na, and 15% methanol at a flow rate of 0.23 mL/min. The samples were manually injected into an analytical column (EICOMPAK SC-5ODS; 2.1 mm × 150 mm; Eicom). Monoamines were electrochemically detected using a graphite electrode (WE-3G; Eicom) at 700 mV relative to a silver/silver chloride reference electrode.

### Statistical analysis

All data values are expressed as the mean ± standard error of mean (SEM). The commercially available IBM SPSS Statistics version 22.0 (IBM, Armonk, USA) software was used for the statistical analysis. Parameters before and after STN-DBS surgery were compared within subjects by Wilcoxon signed-rank test. Statistical significance was set at p < 0.05. Spearman’s rank correlation coefficient was calculated to evaluate the relationship between preoperative catecholamine levels and postoperative motor (UPDRS-II and III score), cognitive (MMSE and FAB), and QOL(PDQ-39 SI and sub scores). A stepwise multiple linear regression study was performed to examine whether post-operative (3 months and 1 year later) motor functions (UPDRS-II and III score in on and off phase) and QOL (PDQ-39 SI) could be influenced by pre-operative plasma and CSF catecholamine levels.

### Standard Protocol Approvals, Registrations, and Patient Consents

The study was approved by Chiba University Hospital Institutional Review Board and all patients gave informed consent. Written informed consent was obtained during on phase from all participants in the study. The approval from an ethical standards committee to conduct this study was received.

None of the patients in this study had a compromised capacity/ability to consent. STN-DBS is contraindication for the patients with cognitive impairments who have a compromised capacity/ability to consent.

## Results

### The effect of STN-DBS on motor functions, quality of life, cognitive function and LED

All procedures were performed without complications. The clinical evaluations of the effects of STN-DBS are listed in Table 1. Mean LED was significantly decreased from 1032 ± 34.6 mg to 620 ± 42.3 mg after 3 months, 588 ±41.2 mg after 1 year, respectively. (Table 1) Furthermore, mean off phase UPDRS-III scores also significantly decreased from 44.3 ±2.3 to 22.4 ± 3.0 after three months, 25.0± 3.7 after 1 year, respectively. (Table 1) Mean PDQ-39 SI also decreased from 33.9 ± 2.9% to 25.6 ± 2.5% after 3 months, 26.7±3.3% after 1 year, respectively. (Table 1) Other clinical scores including UPDRS-II scores, MMSE, FAB are also listed in Table 1.

### The effect of STN-DBS on plasma catecholamine levels

**Fig 1:**
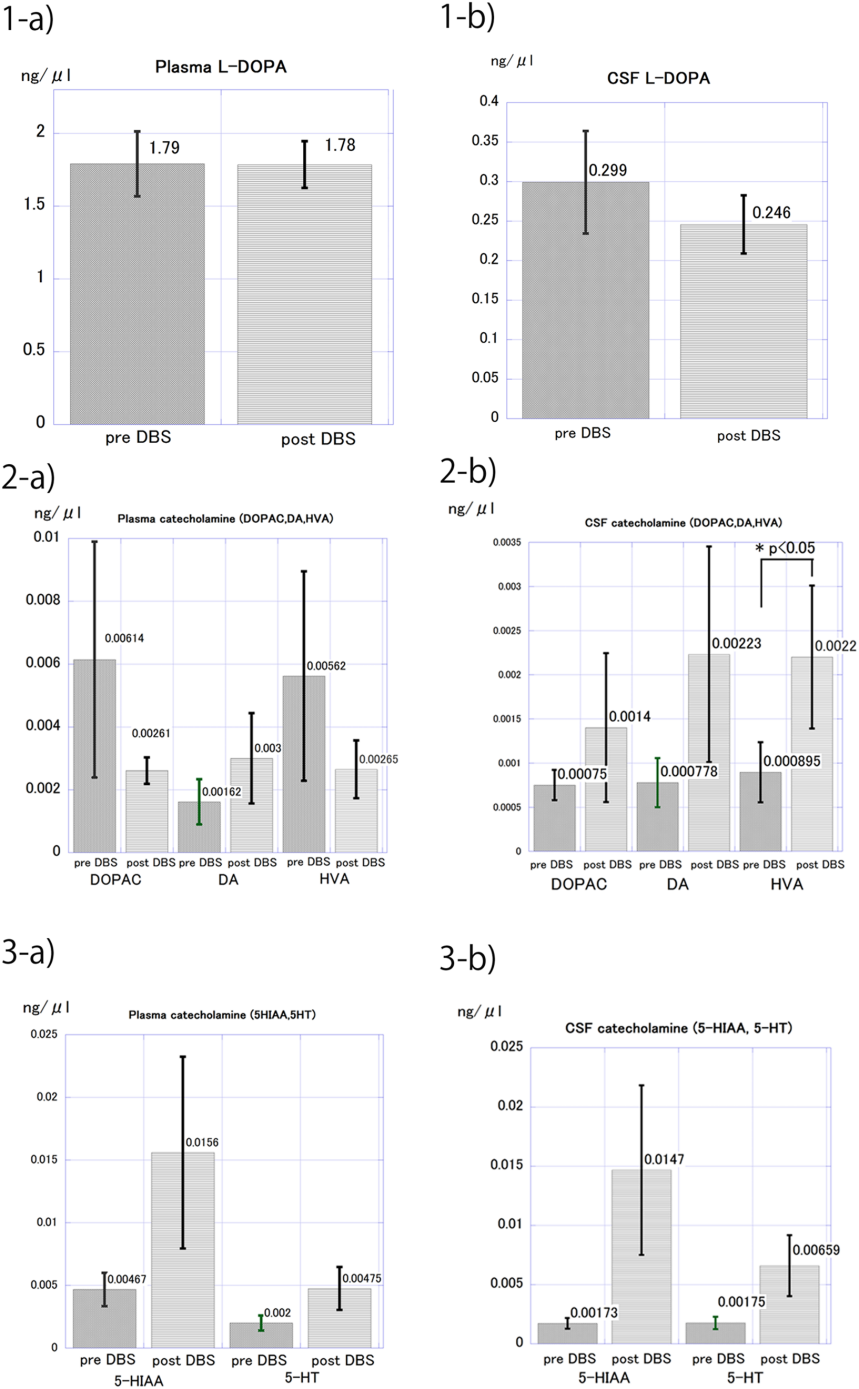
Mean plasma and CSF catecholamine level before and after STN-DBS in 11 patients who were undertaken lumbar puncture before and after STN-DBS. Mean plasma (1-a) and CSF (1-b) levels of L-DOPA were represented. Mean plasma (2-a) and CSF (2-b) levels of catecholamine including DOPAC, DA and HVA were represented. Mean plasma (3-a) and SF (3-b) levels of catecholamine including 5-HIAA and 5-HT were represented. Bar represented SEM.

Of the 25 patients, 11 patients agreed to lumbar puncture procedure at the postoperative follow-up, and postoperative plasma catecholamine levels were measured. Mean LED was significantly decreased from 949 ± 216 mg to 520 ± 315 mg before and after STN-DBS, respectively. Among 8 patients taking entacapone before surgery, 7 stopped its use after surgery. Similarly, among 3 patients taking selegiline before surgery, 2 stopped its use after surgery. Mean plasma L-DOPA levels changed from 1.790 ± 0.222 ng/μl to 1.785 ± 0.160 ng/μl and were not statistically significant. (Fig 1-1-a) Mean plasma DOPAC levels and HVA levels showed large decrease after STN-DBS without statistically significance. Although the change in plasma DOPAC levels and HVA levels were large, the variability indicated as SEM was also large in the preoperative levels of plasma DOPAC and HVA. On the contrary, mean plasma 5-HIAA levels showed large increase after STN-DBS without statistically significance. The variability indicated as SEM was also large in the postoperative levels of 5-HIAA. (Fig 1-2-a and 1-3-a)

### The effect of STN-DBS on CSF catecholamine levels

Of the 20 patients, 11 patients agreed to lumbar puncture procedure at the postoperative follow-up. Mean CSF L-DOPA levels changed from 0.299 ± 0.064 ng/μl to 0.246 ± 0.036 ng/μl and were not statistically significant. (Fig 1-1-b) CSF HVA levels significantly increased from 0.00089±0.0003 ng/μl to 0.002±0.0008 ng/μl after STN-DBS. (Fig 1-2-b) Mean CSF levels of DA, DOPAC, 5-HT and 5-HIAA showed large increase after STN-DBS without statistical significance. The variability indicated as SEM was large in the postoperative levels of CSF DOPAC, DA, 5HIAA and 5-HT. (Fig 1-2-b and 1-2-c)

### The relationship between preoperative catecholamine levels and postoperative motor, cognitive functions and QOL

Some of the preoperative plasma and CSF catecholamine levels had basically negative correlations with postoperative motor functions assessed by UPDRS- III scores suggesting that higher preoperative catecholamine levels were related to better outcome of motor symptoms after STN-DBS.

The preoperative plasma levels of L-DOPA had significantly negative correlations with postoperative UPDRS- III score in off phase three months after STN-DBS, whereas the preoperative plasma levels of HVA had positive correlations with postoperative UPDRS- III score in on and off phase one year after STN-DBS. (Table 2) The preoperative CSF DOPAC and 5-HT levels had significantly negative correlations with postoperative UPDRS- III score in off phase one year after STN-DBS and the preoperative CSF HVA levels had significant negative correlations with postoperative UPDRS- III score in on phase three months after STN-DBS. (Table 3)

**Table 2:** Correlation between preoperative plasma catecholamine levels and motor scores (UPDRS-II and III before and after STN-DBS in on and off phase).

Preoperative plasma catecholamine level		LDOPA pre plasma	DOPAC pre plasma	DA pre plasma	5HIAA pre plasma	HVA pre plasma	5HT pre plasma
**LED pre DBS**	**r**	0.157	-0.298	-0.419	-0.255	-0.088	-0.183
	**p**	0.454	0.189	0.154	0.359	0.706	0.466
**PART2 off 3M after DBS**	**r**	-0.396	0.019	-0.135	-0.455	0.248	-0.365
	**p**	0.228	0.965	0.773	0.305	0.490	0.334
**PART2 off 1Y after DBS**	**r**	0.486	0.800	0.258	0.000	0.500	0.261
	**p**	0.329	0.200	0.742	1.000	0.312	0.618
**PART2 on 3M after DBS**	**r**	-0.078	0.167	0.112	-0.475	0.203	-0.156
	**p**	0.791	0.644	0.792	0.197	0.507	0.628
**PART2 on 1Y after DBS**	**r**	-0.024	0.441	0.000	-0.500	0.200	0.130
	**p**	0.955	0.381	1.000	0.500	0.667	0.758
**PART3 off 3M after DBS**	**r**	-0.508	-0.274	-0.518	-0.412	-0.236	-0.480
	**p**	0.044✤	0.365	0.188	0.310	0.418	0.160
**PART3 off 1Y after DBS**	**r**	0.318	-0.234	0.135	0.174	0.554	-0.279
	**p**	0.290	0.515	0.798	0.742	0.049✤	0.434
**PART3 on 3M after DBS**	**r**	-0.239	-0.454	-0.164	-0.466	-0.014	-0.343
	**p**	0.324	0.089	0.673	0.174	0.956	0.230
**PART3 on 1Y after DBS**	**r**	0.305	-0.078	0.304	-0.174	0.575	0.093
	**p**	0.289	0.821	0.558	0.742	0.039✤	0.786

^✤^ p<0.05.

**Table 3:** Correlation between preoperative CSF catecholamine levels and motor scores (UPDRS-II and III before and after STN-DBS in on and off phase).

Preoperative CSF catecholamine level		LDOPA pre CSF	DOPAC pre CSF	DA pre CSF	5HIAA pre CSF	HVA pre CSF	5HT pre CSF
**LED pre DBS**	**r**	0.129	-0.099	-0.722	-0.305	-0.138	-0.207
	**p**	0.578	0.716	0.028✤	0.361	0.574	0.518
**PART2 off 3M after DBS**	**r**	-0.251	-0.009	-0.057	0.175	-0.274	0.212
	**p**	0.457	0.982	0.904	0.653	0.415	0.614
**PART2 off 1Y after DBS**	**r**	0.143	-0.105	0.105	0.200	-0.617	0.088
	**p**	0.787	0.895	0.895	0.747	0.192	0.868
**PART2 on 3M after DBS**	**r**	-0.201	-0.067	0.127	0.168	-0.491	0.131
	**p**	0.510	0.855	0.765	0.643	0.088	0.717
**PART2 on 1Y after DBS**	**r**	-0.253	-0.054	-0.229	0.200	-0.381	-0.128
	**p**	0.545	0.931	0.711	0.747	0.351	0.784
**PART3 off 3M after DBS**	**r**	-0.343	-0.349	-0.227	-0.006	-0.326	0.139
	**p**	0.193	0.243	0.589	0.986	0.235	0.701
**PART3 off 1Y after DBS**	**r**	0.254	-0.730	-0.278	0.441	-0.239	-0.757
	**p**	0.426	0.039✤	0.594	0.381	0.454	0.018*
**PART3 on 3M after DBS**	**r**	-0.234	-0.055	0.009	-0.338	-0.638	-0.179
	**p**	0.350	0.857	0.982	0.340	0.004✤	0.579
**PART3 on 1Y after DBS**	**r**	-0.078	-0.276	0.123	0.353	-0.273	-0.497
	**p**	0.800	0.509	0.816	0.493	0.366	0.144

^✤^ p<0.05.

With regard to cognitive functions only preoperative CSF levels of DA had significantly negative correlations with postoperative FAB scores three months after STN-DBS. (Tables 4 and 5) In PDQ-39 SI, preoperative plasma DA level had significantly negative correlations with postoperative PDQ-39 SI one year after STN-DBS suggesting that higher preoperative plasma DA level resulted in better QOL one year after STN-DBS. Some of the preoperative levels of both plasma and CSF catecholamine had significantly negative correlations with pre and postoperative sub-scores of PDQ-39. (Tables 6 and 7)

**Table 4:** Correlation between preoperative plasma catecholamine levels and cognitive function (MMSE and FAB) before and after (3 months and 1 year) STN-DBS.

Preoperative plasma catecholamine level		LDOPA pre plasma	DOPAC pre plasma	DA pre plasma	5HIAA pre plasma	HVA pre plasma	5HT pre plasma
**MMSE 3M after DBS**	**r**	0.108	-0.095	-0.117	-0.048	-0.418	0.272
	**p**	0.702	0.757	0.783	0.903	0.155	0.392
**MMSE 1Y after DBS**	**r**	-0.218	-0.067	-0.447	0.526	0.113	0.111
	**p**	0.604	0.887	0.450	0.362	0.809	0.813
**FAB 3M after DBS**	**r**	0.269	-0.145	-0.280	-0.173	0.389	-0.461
	**p**	0.423	0.709	0.543	0.710	0.300	0.212
**FAB 1Y after DBS**	**r**	-0.514	0.121	0.000	-0.707	0.045	-0.269
	**p**	0.238	0.819	1.000	0.293	0.933	0.607

^✤^ p<0.05.

**Table 5:** Correlation between preoperative CSF catecholamine levels and cognitive function (MMSE and FAB) before and after (3 months and 1 year) STN-DBS.

Preoperative CSF catecholamine level		LDOPA pre CSF	DOPAC pre CSF	DA pre CSF	5HIAA pre CSF	HVA pre CSF	5HT pre CSF
**MMSE 3M after DBS**	**r**	0.043	0.071	-0.010	-0.313	-0.003	0.312
	**p**	0.884	0.845	0.983	0.413	0.993	0.380
**MMSE 1Y after DBS**	**r**	-0.691	-0.592	-0.211	-0.308	-0.297	0.088
	**p**	0.086	0.293	0.789	0.614	0.567	0.868
**FAB 3M after DBS**	**r**	0.292	-0.189	-0.850	0.045	0.276	-0.458
	**p**	0.383	0.653	0.032✤	0.915	0.411	0.254
**FAB 1Y after DBS**	**r**	-0.618	-0.544	-0.500	-0.949	-0.730	0.289
	**p**	0.191	0.456	0.667	0.051	0.161	0.637

^✤^ p<0.05.

**Table 6:** Correlation between preoperative plasma catecholamine levels and PDQ-39 sub-score before and after (3 months and 1 year) STN-DBS.

		LDOPA pre plasma	DOPAC pre plasma	DA pre plasma	5HIAA pre plasma	HVA pre plasma	5HT pre plasma
**PDQ-39SI 3M**	**r**	-0.145	-0.137	-0.518	-0.515	-0.217	-0.113
	**p**	0.607	0.655	0.188	0.156	0.477	0.755
**PDQ-39SI 1Y**	**r**	-0.382	-0.211	-0.894	-0.154	-0.499	-0.434
	**p**	0.247	0.586	0.040✤	0.805	0.142	0.283
**mobility 3M**	**r**	0.114	0.077	-0.261	-0.339	-0.216	0.088
	**p**	0.685	0.802	0.533	0.372	0.479	0.808
**mobility 1Y**	**r**	0.291	-0.284	-0.671	-0.616	0.112	-0.265
	**p**	0.386	0.458	0.215	0.269	0.758	0.526
**ADL 3M**	**r**	0.169	-0.147	-0.127	-0.552	-0.151	0.202
	**p**	0.547	0.632	0.765	0.124	0.624	0.576
**ADL 1Y**	**r**	0.132	-0.310	-0.447	-0.616	-0.003	-0.590
	**p**	0.698	0.417	0.450	0.269	0.993	0.123
**emotional well being 3M**	**r**	-0.362	-0.006	-0.192	-0.567	-0.105	-0.032
	**p**	0.185	0.985	0.649	0.112	0.734	0.931
**emotional well being 1Y**	**r**	-0.543	-0.005	-0.224	-0.526	-0.747	-0.091
	**p**	0.084	0.991	0.718	0.362	0.013✤	0.831
**stigma 3M**	**r**	-0.011	0.163	-0.351	-0.049	-0.239	0.107
	**p**	0.969	0.594	0.394	0.901	0.432	0.768
**stigma 1Y**	**r**	-0.387	-0.038	-0.344	0.158	-0.690	-0.171
	**p**	0.240	0.923	0.571	0.800	0.027✤	0.686
**social support 3M**	**r**	-0.154	-0.003	-0.338	-0.549	0.099	0.003
	**p**	0.583	0.992	0.414	0.126	0.748	0.993
**social support 1Y**	**r**	-0.270	0.116	-0.646	0.181	-0.316	0.158
	**p**	0.423	0.766	0.239	0.770	0.374	0.708
**cognition 3M**	**r**	-0.288	-0.088	0.000	-0.504	-0.378	-0.153
	**p**	0.298	0.776	1.000	0.166	0.203	0.672
**cognition 1Y**	**r**	-0.482	-0.186	-0.918	0.158	-0.418	-0.073
	**p**	0.134	0.632	0.028✤	0.800	0.229	0.863
**communication 3M**	**r**	-0.165	-0.200	-0.633	-0.468	-0.339	-0.173
	**p**	0.557	0.512	0.092	0.204	0.257	0.633
**communication 1Y**	**r**	-0.380	-0.019	-0.631	0.632	-0.161	-0.224
	**p**	0.249	0.962	0.254	0.253	0.656	0.594
**bodily discomfort3M**	**r**	-0.140	-0.251	-0.097	0.306	0.239	-0.274
	**p**	0.619	0.407	0.820	0.423	0.433	0.444
**bodily discomfort1Y**	**r**	-0.367	-0.498	-0.894	0.000	-0.189	-0.580
	**p**	0.267	0.173	0.040✤	1.000	0.600	0.132

^✤^ p<0.05.

**Table 7:** Correlation between preoperative CSF catecholamine levels and PDQ-39 sub-score before and after (3 months and 1 year) STN-DBS.

		LDOPA pre CSF	DOPAC pre CSF	DA pre CSF	5HIAA pre CSF	HVA pre CSF	5HT pre CSF
**PDQ-39SI 3M**	**r**	-0.295	0.107	-0.374	-0.580	-0.268	0.035
	**p**	0.286	0.728	0.321	0.102	0.354	0.928
**PDQ-39SI 1Y**	**r**	-0.261	-0.621	-0.949	-0.821	-0.367	-0.048
	**p**	0.467	0.188	0.051	0.089	0.331	0.909
**mobility 3M**	**r**	0.007	0.285	-0.063	-0.459	0.186	0.187
	**p**	0.980	0.346	0.873	0.214	0.525	0.630
**mobility 1Y**	**r**	0.418	-0.207	-0.632	0.359	0.578	-0.655
	**p**	0.229	0.694	0.368	0.553	0.103	0.078
**ADL 3M**	**r**	0.178	0.221	0.174	-0.120	-0.203	0.067
	**p**	0.526	0.469	0.655	0.758	0.487	0.863
**ADL 1Y**	**r**	0.280	-0.105	-0.632	-0.205	0.461	-0.390
	**p**	0.433	0.843	0.368	0.741	0.212	0.339
**emotional well being 3M**	**r**	-0.509	-0.083	-0.107	-0.241	-0.601	0.049
	**p**	0.052	0.787	0.783	0.532	0.023✤	0.900
**emotional well being 1Y**	**r**	-0.541	-0.210	-0.316	-0.975	-0.486	0.327
	**p**	0.106	0.690	0.684	0.005	0.184	0.429
**stigma 3M**	**r**	-0.246	0.303	-0.201	-0.537	0.018	0.357
	**p**	0.376	0.315	0.604	0.136	0.951	0.346
**stigma 1Y**	**r**	-0.246	-0.746	-1.000	-0.763	-0.084	0.099
	**p**	0.493	0.088	<.0001	0.133	0.830	0.815
**social support 3M**	**r**	-0.400	0.042	-0.406	-0.051	-0.069	0.091
	**p**	0.140	0.891	0.279	0.896	0.814	0.816
**social support 1Y**	**r**	-0.475	-0.490	-0.544	-0.740	-0.570	0.357
	**p**	0.165	0.324	0.456	0.152	0.109	0.386
**cognition 3M**	**r**	-0.196	0.062	0.238	-0.291	-0.603	-0.222
	**p**	0.485	0.841	0.537	0.447	0.022✤	0.566
**cognition 1Y**	**r**	-0.379	-0.533	-1.000	-0.763	-0.523	0.331
	**p**	0.280	0.276	<.0001	0.133	0.149	0.423
**communication 3M**	**r**	-0.261	-0.234	-0.649	-0.281	-0.272	-0.193
	**p**	0.348	0.441	0.059	0.463	0.348	0.619
**communication 1Y**	**r**	-0.339	-0.735	-0.738	-0.564	-0.518	0.215
	**p**	0.339	0.096	0.262	0.322	0.153	0.610
**bodily discomfort3M**	**r**	-0.069	-0.389	-0.356	0.054	-0.222	-0.453
	**p**	0.807	0.189	0.347	0.891	0.445	0.220
**bodily discomfort1Y**	**r**	-0.018	-0.621	-0.949	-0.205	-0.106	-0.427
	**p**	0.960	0.188	0.051	0.741	0.786	0.292

^✤^ p<0.05.

### Stepwise multiple linear regression analysis

In UPDRS-II, both on and off score three months after STN-DBS was positively influenced by preoperative plasma HVA levels, whereas both on and off score 1 year after STN-DBS was not influenced by any of preoperative plasma and CSF catecholamine levels. (Table 8)

**Table 8:** Stepwise multiple linear regression study.

Dependent variables	Explanatory variables	standardized β	p value
Part2 off score 3M after DBS	pre plasma HVA	0.972	p = 0.005
Part2 off score 1Y after DBS	none		
Part2 on score 3M after DBS	pre plasma HVA	0.882	p = 0.02
Part2 on score 1Y after DBS	none		
Part3 off score 3M after DBS	pre plasma HVA	0.985	p<0.0001
Part3 off score 1Y after DBS	pre plasma HVA	0.866	p = 0.011
	pre CSF L-DOPA	-0.233	p = 0.040
Part3 on score 3M after DBS	pre CSF L-DOPA	-0.581	p = 0.038
	pre plasma HVA	0.528	p = 0.048
Part3 on score 1Y after DBS	none		
PDQ 39 SI 3M after DBS	pre CSF L-DOPA	-0.87	p = 0.024
PDQ 39 SI 1Y after DBS	pre CSF DA	-0.996	p = 0.004

In UPDRS- III, off score three months after STN-DBS was positively influenced by preoperative plasma HVA levels, whereas off score 1 year after STN-DBS was positively influenced by preoperative plasma HVA levels and negatively influenced by preoperative CSF L-DOPA levels. On the other hand, UPDRS- III on score three months after STN-DBS was negatively influenced by preoperative CSF L-DOPA levels and positively influenced by plasma HVA levels, whereas on score one year after STN-DBS was not influenced by any of preoperative plasma and CSF catecholamine levels. (Table 8)

In PDQ-39 SI, QOL three months after STN-DBS was negatively influenced by preoperative CSF L-DOPA levels, whereas QOL 1 year after STN-DBS was negatively influenced by preoperative CSF DA levels. (Table 8)

## Discussion

The present study revealed that preoperative plasma and CSF catecholamine levels had significant correlations with postoperative motor, cognitive functions and QOL. Basically, the higher catecholamine levels were correlated with better motor, cognitive functions and QOL postoperatively with a few exceptions.

Stepwise multiple linear regression study showed that preoperative plasma HVA levels positively influenced UPDRS part IIand part III score after STN-DBS, whereas preoperative CSF L-DOPA levels negatively influenced UPDRS part II and part III score and PDQ-39 SI after STN-DBS. These results suggested that higher preoperative plasma HVA levels had negative influence on the postoperative motor symptoms (i.e., increase in the score of UPDRS), which is difficult to understand. On the other hand higher preoperative CSF L-DOPA levels had positive influence on the postoperative motor symptoms and QOL (decrease in the score of UPDRS and PDQ-39 SI), which seemed to be compatible with the notion that good responsiveness to L-DOPA therapy predict better outcome after STN-DBS [1].

The present study also examined how plasma and CSF levels of catecholamine change after STN-DBS. Although, mean plasma levels of DOPAC and HVA showed large decrease after STN-DBS without statistical significance, mean CSF levels of HVA showed large increase after STN-DBS with statistically significance despite the marked reduction in mean LED after STN-DBS. Mean CSF levels of DA and DOPAC showed large increase after STN-DBS without statistically significance. Mean plasma levels of 5-HIAA and CSF levels of 5-HIAA and 5-HT showed large increase without statistically significance.

The precise effects of STN-DBS on catecholamine levels remain unclear with studies finding STN-DBS to increase, decrease, or maintain striatal monoamine levels in normal and PD rat models [8,9,11–14,17]. In addition, we have previously reported that the striatal levels of DOPAC were significantly decreased after cessation of subthalamic stimulation in PD rat models in which 6-hydroxydopamine was injected into left medial forebrain bundle [18]. In humans, positron emission tomography (PET) measurements of [^11^C] raclopride uptake during STN-DBS failed to demonstrate any change in striatal DA [10].

Although the present study revealed that CSF catecholamine levels tended to increase after STN-DBS despite the significant reduction in LED, it might be difficult to draw any conclusions due to small number of patients and large variability in SEM. We also do not know why plasma levels of DOPAC and HVA tended to decrease after STN-DBS. One of the advantage effects of the decreased plasma levels of DOPAC and HVA might be neuroprotection for PD. DOPAC and HVA are neurotoxic and induce apoptosis in cultured dopaminergic neuron by augmenting free radical-induced mitochondrial degeneration and eventually augmenting Charnoly Body (CB) formation implicated in apoptosis and progressive neurodegeneration. Metallothioneins (MTs) are low molecular weight (6-7kDa) cysteine-rich and antioxidant proteins that provide neuroprotection. In neurodegenerative disease, MTs inhibit CB formation by serving as free radical scavengers [19–22]. MTs might also inhibit the neurotoxic effects of DOPAC and HVA. STN-DBS may augment brain regional MTs to provide mitochondrial neuroprotection in PD which remains to be further explored. However, these points need to be investigated with larger number of patients.

The correlations between preoperative catecholamine levels and postoperative clinical outcome have never been evaluated. However, because clinical outcome of STN-DBS depend on the responsiveness of preoperative L-DOPA treatment [1], preoperative catecholamine levels might be important for the prediction of clinical outcome of STN-DBS. The present results were basically compatible with the predictions that higher levels of plasma and CSF catecholamine were significantly correlated with better motor functions (decrease in UPDRS-III scores), cognitive functions (increase in FAB scores) and better QOL (decrease in PDQ-39 SI and its sub-scores) postoperatively. However, the preoperative plasma HVA levels showed significantly positive correlations with the postoperative UPDRS-III score during the on and off phase examined one year after STN-DBS, whereas preoperative CSF HVA levels showed significantly negative correlations with postoperative UPDRS-III scores during on phase examined three months after STN-DBS. These contradictory results are difficult to understand.

CSF levels of 5-HT had negative correlations with postoperative UPDRS- III scores. This result suggest that higher levels of CSF 5-HT might be correlated with better motor functions postoperatively. Although, there are a large number of studies examining the levels of 5-HT and 5-HIAA with relation to motor and psychiatric symptoms in animal model of PD and patients with PD [15, 23], the results of these studies were controversial. The present results concerning the preoperative CSF levels of 5-HT and postoperative clinical outcome should be evaluated in more detail in the future.

A stepwise multiple linear regression analysis revealed that higher preoperative plasma HVA levels had negative influence on the postoperative motor symptoms, whereas higher preoperative CSF L-DOPA levels had positive influence on the postoperative motor symptoms and QOL. The higher preoperative CSF DA levels also had positive influence on the QOL one year after STN-DBS. Although it seemed to be reasonable that higher preoperative CSF L-DOPA and DA levels had positive influence on the postoperative motor symptoms and QOL, it is hard to understand that higher preoperative plasma HVA levels had negative influence on the postoperative motor symptoms. Because plasma HVA are derived from extra-neuronal organs such as liver, kidney and mesenteric organs containing catechol-O-methyltransferase [24], it might be difficult to relate the plasma HVA levels with clinical symptoms in PD. On the contrary, several previous studies discussed the relationship between CSF HVA levels and clinical symptoms in PD [23, 25]. However, increased CSF HVA levels caused by L-DOPA treatment was not related with clinical improvement according to previous studies [23]. The relationships between CSF HVA levels and clinical symptoms need to be more carefully investigated in the future to conclude that whether preoperative CSF HVA levels are helpful for predicting postoperative clinical outcome.

There are some limitations to the present results. We could examine the postoperative CSF catecholamine levels in only 11 patients in this study. The small number of patients might have biased the results of postoperative CSF catecholamine levels in this study. The other limitation was that we did not measure the CSF catecholamine level during off stimulation, which might be important to confirm the effect of STN-DBS on CSF catecholamine levels. Stimulation should be turned off approximately 12 hours before lumbar puncture to evaluate the CSF catecholamine levels during stimulation off states. However, it is technically difficult to perform lumbar puncture during off stimulation due to the marked axial rigidity and bradykinesia. The patients might be suffering from keeping lateral position for more than 10 minutes during off stimulation. It seems to be unrealistic to perform lumbar puncture during off stimulation.

Another limitation in this study was that plasma and CSF levels of catecholamine were only assessed at 1 point before and after STN-DBS; therefore we do not know the extent to which the present data reflect overall catecholamine levels.

Although the AUC (Area under the curve) of CSF catecholamine may be more suitable for assessment than a 1-point evaluation of CSF catecholamine [26–28], it not only requires invasive procedures, but it is unrealistic to monitor CSF catecholamine levels for a few hours before and after STN-DBS in large numbers of patients.

## Conclusion

The preoperative catecholamine levels might affect the postoperative motor symptoms and quality of life. The catecholamine levels were not significantly reduced postoperatively despite the significant reduction in levodopa equivalent doses.
